# The Stomach

**Published:** 1870-01

**Authors:** 


### THE STOMACH.

**Familiarly Treated.**

BY ISHMAEL.

When some one asked a famous doctor if it would be healthy to take a walk upon an empty stomach, his pertinent reply was, that it depended very much upon whose stomach his questioner proposed to walk.

This is what I call treating the stomach in a familiar manner, in a manner which all can understand, and it is in something of this style that I propose to continue my article, which was barely introduced in the last number of this Quarterly. Barely, but not nakedly, for it was decently clothed in as pleasant language as I could command. To be accurate, however, it is scarcely proper to say that a or the stomach was introduced into a BISTOURY. A Bistoury could readily be introduced into a stomach, but hardly could a stomach be introduced into a Bistoury. To see the excellence of the last sentence, it is perhaps necessary to say, for the benefit of those whose medical education has been neglected, that *a* Bistoury is unlike THE BISTOURY, simply in the number of its points; a Bistoury being a sharp surgical instrument with *one* fine point, while THE BISTOURY has many.

I assure my readers that I speak as one having authority, for I had the honor of standing sponsor when this journal was christened.

To return to our stomach, as the man said who had made three unsuccessful attempts at swallowing an oyster, and was trying it for the fourth time; to return to our stomach. We have already learned its shape, of what it is composed, how it does its work, and the name of its one help-meet or servant with whose aid it digests the food sent down to it, this one, active, almost intelligent servant called Gastric Juice.

G. J. is an exceedingly powerful liquid and has performed many a wonderful trick. For instance, let one lose his favorite, bone handled, four-bladed pocket knife in October, and let him be fortuate enough to find it again on the succeeding April, six months thereafter. It has been rained on, blown on, and mayhap has lain under the snow during the winter months of the time it is lost, and when recovered appears rusty and old. Brush off the rust, rub the blades over your boot-leg a few times and there you have your knife, but little the worse for its forced out-door life. The action of the elements oxidizing the knife, would of course in time have destroyed it, but it would have taken a long number of years. Now if the knife had been lost in the recesses of some capacious stomach,the action of the gastric juice would have destroyed it in less time than the elements to which it had been exposed. For, attend. The gastric juice of a dog will dissolve ivory itself, and even the enamel of the teeth; that of the hen dissolved an onyx and diminished a gold coin, while not long since a post-mortem examination was held on the body of a man who died in one of the public hospitals in London. Some three years before, he had swallowed, (he was the original knife-swallower,) a number of pocket knives, the handles of which were found to be entirely digested, and the blades more than three-quarters diminished! Not a very nutritious diet, to be sure, and one not at all likely to become fashionable, but one which makes quite reasonable the sneer hurled at would-be wits and humorists, that they have been eating razors!

However much it my be regretted by gourmands and practiced diners-out, it is nevertheless true, that man is inferior to some other animals, in that while he has only one stomach, there are other creatures, like the ostrich, that have two stomachs, like the kangaroo, that have three stomachs, or more still, like the ox, the sheep and the sloth, that have four stomachs.

I heard a man once, while tasting a delicious bit of cookery, wish that he had a neck like a giraffe, that he might the longer retain the grateful flavor that tickled his palate; what would have been his sentiments had he been aware of the fact just above stated?

Of course, there are stomachs, and—stomachs. Each kind for its own peculiar labor and duty, refusing to do other kinds. There is, for instance, the stomach of the goose, the turkey or the hen, commonly called, and eagerly devoured by children at Thanksgiving and holiday-time under the name of the gizzard. Gizzard is a bad word and a hard, but it is no harder than the work that it is capable of doing. With the ease of a mill-stone, it literally grinds to powder pebbles, small rocks, large pieces of gravel, and seems never to be satisfied unless it has constantly on hand a small sand bank. Mr. J. HUNTER, a distinguished physiologist, but in whose count I don't have much faith, mentioning his name for that reason, sat up one night and counted the number of pebbles in the gizzard of a turkey, and also in that of a goose; in the first he found two hundred, and in the latter one thousand!

It may be observed by the skeptical at this point, that either the pebbles were small, or else the gizzard very large,—much larger than mine, at best, and I only know of one stomach that would at all compare with such a carrying capacity. It is that one mentioned in Othello, and of course my intelligent reader will require no closer reference.*

Some cruel men too, the further experimenting on the strength of the gizzard in a fowl, compelled some geese and other birds, I think they were all geese, to swallow pins and needles, the blades of lancets, tacks and other hard and pointed substances. They had to kill the birds after all, for they were perfectly at home on this diet, and on examining the gizzard, they were found to have sustained no injury at all, but the substances swallowed were broken and otherwise maltreated. The Ostrich too, takes into his gizzard, pebbles and sand, literally doing what the old song accuses him of, namely, eating cobble-stones!

On the other extreme, there are animals who have such a delicate or perhaps accomodating stomach, that they can live on water or air or nothing. Leeches and tadpoles unfortunately for mankind, live quite comfortably on the former, and the silver and gold fish, with the pike, even, the most gluttonous of the whole class, have been known to both live and thrive for years upon water alone in a marble basin. For those who live upon air, we have the snail and the chameleon; a spider has been known to do it too, on a pinch, or rather when stuck to a cork,—and toads, shut up in rocks, for centuries, where neither water nor air can reach them, have hopped out from their cells when released, thin and hungry, perhaps, but lively.—Of other examples of those who live upon nothing, I can call to mind only two more, the unfortunates at the Poor House and the family of a ruined Banker.

A folio of interesting matter might be written concerning the subject I have under consideration, but as I propose to treat other portions of the human body in much the same manner, I will close up on the stomach, feeling satisfied that I have accorded to it, the place it occupies everywhere, and that is, that it goes first with everybody.

---

*Had all his hairs been lives, my great revenge

Had stomach for them all.

OTHELLO Act V.

